# Pipelle for Pregnancy (PIP): study protocols for three randomised controlled trials

**DOI:** 10.1186/s13063-016-1301-9

**Published:** 2016-04-27

**Authors:** Sarah Lensen, Wellington Martins, Carolina Nastri, Lynn Sadler, Cindy Farquhar

**Affiliations:** Department of Obstetrics and Gynaecology, University of Auckland, Auckland, New Zealand; Department of Obstetrics and Gynecology, Medical School of Ribeirao Preto, University of Sao Paulo, Ribeirao Preto, Brazil

**Keywords:** Endometrial injury, Endometrial scratching, Endometrial receptivity, Inflammation, Implantation

## Abstract

**Background:**

The success rate of infertility treatments remains modest. Endometrial injury has been suggested as an intervention to increase the probability of pregnancy in women undergoing assisted reproductive technologies such as in vitro fertilisation (IVF). The majority of studies and systematic reviews have reported that endometrial injury improves the outcome**s** of IVF, intrauterine insemination and natural conception; however, the size and quality of the studies are poor. The low quality of the available evidence questions the presence of any real beneficial effect, and the applicability of the intervention in different populations remains unclear.

**Methods/design:**

The PIP trials are three multi-centre, randomised controlled trials designed to test three separate hypotheses: whether endometrial injury increases the probability of live birth in women or couples

1) who are undergoing autologous embryo transfer as part of an IVF cycle (PIP-IVF),

2) with unexplained infertility who are attempting to conceive naturally (PIP-UE) and

3) with subfertility related to polycystic ovarian syndrome (PCOS) who are on ovulation induction medication and attempting to conceive (PIP-PCOS).

Participants will be randomised to either undergo endometrial injury by endometrial pipelle biopsy or to:

• no intervention (PIP-IVF), or

• a sham procedure (PIP-PCOS and PIP-UE).

In PIP-IVF, endometrial injury will be carried out between day three of the cycle prior to the IVF cycle, and day three of the IVF cycle. In PIP-UE and PIP-PCOS, endometrial injury or a sham procedure will be undertaken between days 1–12 of a menstrual cycle or ovulation induction cycle respectively. Participants in PIP-UE and PIP-PCOS will then be followed for three cycles during which time they will attempt to conceive from sexual intercourse. To ensure allocation concealment, randomisation will be carried out using a web-based system or sequentially numbered, opaque, sealed envelopes. The primary outcome is live birth. Secondary outcomes include ongoing pregnancy, clinical pregnancy and miscarriage.

The required sample sizes for the PIP studies have been estimated at 840 (PIP-IVF), 350 (PIP-UE) and 280 (PIP-PCOS). Primary analysis will be as per intention-to-treat principles.

**Discussion:**

The PIP trials are designed to address the gaps in the utility of endometrial scratching as a treatment for subfertility in three different populations. If the beneficial effect of this intervention can be confirmed in these settings, endometrial scratching will provide a cost-effective method for helping women and couples to conceive.

**Trial registrations:**

PIP-IVF ACTRN12614000626662 registered 13/6/2014; PIP-PCOS ACTRN12614000657628 registered 24/6/2014; PIP-UE ACTRN12614000656639 registered 24/6/2014. The trials are ongoing.

## Background

Having a baby is an important goal for many couples. Unfortunately, up to 10 % of couples will not achieve pregnancy after a year of frequent, unprotected intercourse and are defined as being subfertile [1]. Many subfertile couples seek fertility treatments to help them conceive. However, even the most successful fertility treatment, in vitro fertilisation (IVF), has a live birth rate of only 30 % per cycle [2, 3]. Failure of an IVF cycle to result in pregnancy can be due to a number of factors and, even when high quality embryos are replaced, implantation rates remain modest. A lack of adequate endometrial receptivity is thought to be responsible for up to two-thirds of unsuccessful embryo transfers [4].

Endometrial injury is currently being proposed as a technique to improve the endometrial receptivity and in turn the probability of conception in IVF cycles. Endometrial biopsy is the most common procedure used to deliver an endometrial injury [5]. The procedure involves the insertion of a catheter, such as a pipelle sampler, through the cervix and into the uterus, where a sample of the endometrium is obtained via light suction and rotation within the uterine cavity. The biopsy results in a ‘scratch’ or ‘injury’ to the endometrium during the process of obtaining a sample. Outside of the fertility area, pipelle biopsy is routinely performed to collect endometrial samples for diagnostic purposes, for example, in women with abnormal menstrual bleeding. It is a well-tolerated procedure with a proven safety profile [6, 7].

In 2000 it was first reported in a retrospective study that 11 of 12 women who had undergone a series of investigational endometrial biopsies conceived in their subsequent IVF cycle [8]. A prospective non-randomised study by the same research group reported that women allocated to endometrial biopsy in the cycle prior to IVF cycle were twice as likely to conceive as women who had not [9]. Several studies have since investigated the apparent beneficial effect of endometrial injury in IVF cycles, and although there is substantial heterogeneity in the study designs and participant populations, a recent Cochrane review including randomised controlled trials (RCTs) reports an increase in the probability of pregnancy and live birth following endometrial sampling in the cycle preceding embryo transfer (RR = 1.34, 95 % CI 1.21–1.61 and RR = 1.42, 95 % CI 1.08–1.85, respectively) [5]. Other reviews and meta-analyses which have included both RCTs and non-randomised studies have similar conclusions [10–12]. The effect appears to be the most pronounced in women who have repeatedly failed to achieve a pregnancy after multiple embryo transfers, that is, women with recurrent implantation failure (RIF) [13]. Note that in the only study where endometrial injury was performed on the day of oocyte retrieval, a detrimental effect on pregnancy rate was observed [14].

Almost all of the RCTs assessing endometrial injury are associated with significant limitations that place them at a high risk of bias, which is known to be associated with exaggerated effect estimates (such as lack of adequate blinding and allocation concealment, high rates of participant attrition), or raise questions regarding the generalisability of results (such as use of multiple embryo transfer policies or ovulation induction medications in couples with unexplained infertility, timing of endometrial injury, and the device used for causing endometrial injury). Another common issue in a number of the studies is the potential exposure of the control group to unintentional endometrial injury resulting from a placebo procedure [15–17] or a co-intervention such as hysteroscopy, either prior to study enrolment [18] or simultaneously with the study procedure [19, 20]. Finally, only a small proportion of the studies reported live birth, arguably the most important outcome for both clinicians and subfertile couples.

The mechanism by which endometrial injury may increase the probability of conception has been the topic of a number of studies and speculations, and is extensively summarised in recent reviews [10, 21]. The most frequently cited theory is that of inflammation. Endometrial scratching has been shown to induce an inflammatory response in the endometrium, which is believed to mediate the dialogue between the endometrium and the blastocyst and facilitate the process of implantation [22]. The resultant inflammatory response involves the production of inflammatory cytokines and subsequent recruitment of macrophages and other immune cells, factors known to be present during the window of implantation. This hypothesis is supported by repeat biopsy studies, in which women who had undergone a second biopsy had increased levels of cytokines such as TNF-α and MIP-1B, and other molecules known to be associated with implantation, such as osteopontin [22–25]. It is possible that endometrial injury may also be beneficial in women with unexplained infertility or polycystic ovarian syndrome (PCOS) who are trying to conceive from sexual intercourse; conditions of infertility in which poor endometrial receptivity has been implicated [26–30].

Due to the generally poor quality of the published studies and a lack of a clear understanding regarding the underlying mechanism of effect, the reproductive benefit of endometrial injury remains controversial [5, 31–34]. This protocol describes studies to be undertaken in three separate groups of women in whom inadequate endometrial receptivity may be partly responsible for the experienced infertility: unexplained infertility, PCOS and women undergoing embryo transfer as part of an IVF/intracytoplasmic sperm injection (ICSI) cycle.

Endometrial biopsy is a simple, cheap and well-tolerated procedure which can be performed without anaesthetic in the out-patient setting [6, 7]. Endometrial biopsy may be a cost-effective method for increasing the probability of pregnancy and live birth in women or couples who are undergoing IVF or who are trying to conceive naturally or with ovulation induction.

### Research questions

The Pipelle for Pregnancy (PIP) studies will evaluate whether endometrial injury delivered by an endometrial pipelle biopsy procedure increases the probability of live birth in

1. Women undergoing autologous embryo transfer; a fresh transfer as part of an IVF cycle or a frozen-thaw embryo transfer (PIP-IVF)
2. Couples with unexplained infertility attempting to conceive spontaneously (PIP-UE)
3. Couples with subfertility associated with polycystic ovarian syndrome who are on ovulation induction medication and trying to conceive (PIP-PCOS).

## Methods/design

The PIP studies are pragmatic, parallel, multi-centre, superiority RCTs.

The pragmatic nature of the studies is a result of the broad inclusion criteria, time frame in which the intervention is administered and lack of restriction to specific stimulation or ovulation induction protocols. This rationale is based on the existing evidence in favour of endometrial scratching before IVF, in which a benefit has been shown across a range of time frames for performing the intervention and in different patient populations. Inclusion of a heterogeneous population in a large study provides an opportunity to detect treatment effects within different patient subgroups.

### Blinding

PIP-IVF is an open label study; women will be aware of their study allocation as they will be randomised to undergo either endometrial biopsy or no procedure.

PIP-PCOS and PIP-UE are single-blind studies; women will not be aware of their study allocations as they will be randomised to either an endometrial biopsy or a sham procedure (placement of the pipelle in the anterior fornix).

In all three studies, the doctor performing the procedure will necessarily be aware of the participant’s allocation.

### Outcomes

The primary outcome is live birth rate per woman randomised, as defined previously [35].

Secondary outcomes include:

- ongoing pregnancy per woman randomised (defined as continuing pregnancy diagnosed by ultrasonographic visualisation of one or more gestational sacs on or after 12 weeks gestation)
- clinical pregnancy per women randomised (defined as a pregnancy diagnosed by ultrasonographic visualisation of one or more gestational sacs, including ectopic pregnancy)
- biochemical pregnancy (defined as βhcg > 25 mIU/ml) PIP-IVF only
- multiple pregnancies per women randomised and per clinical pregnancy (defined as ultrasonographic visualisation of two or more gestational sacs or heartbeats)
- ectopic pregnancy per women randomised and per clinical pregnancy (defined as a pregnancy in which implantation takes place outside the uterine cavity)
- miscarriage per women randomised and per clinical pregnancy (defined as the spontaneous loss of a clinical pregnancy before 20 completed weeks of gestational age)
- neonatal characteristics (for example, gender, gestation at birth, malformations)
- placenta characteristics (placenta previa: major, placenta previa: minor, placental abruption, placenta accreta: previous uterine surgery, placenta accreta: no previous uterine surgery)
- pain during the procedure (as measured by a 10 cm visual analogue scale (VAS))
- bleeding the day following the procedure.

### Eligibility criteria

#### Inclusion criteria

Women who meet the following inclusion criteria are eligible to take part in the PIP studies:

##### PIP-IVF

1. Planning to undergo autologous embryo transfer

Fertility clinics that routinely perform single embryo transfers are eligible to recruit for the PIP-IVF study. As per the pragmatic nature of the PIP studies, a number of women undergoing double embryo transfers will be recruited. The proportion of women in PIP-IVF who are undergoing double embryo transfers should not exceed the normal proportion of women who undergo double embryo transfers at the participating clinics (no more than 20 % [3]).

##### PIP-UE

1. In a relationship where the couple is having regular, unprotected sexual intercourse and pregnancy is desired
2. 42 years of age or younger at the time of randomisation
3. Diagnosed with unexplained infertility, must meet all of:
  - Unsuccessfully trying to conceive for at least 12 months,
  - Normal ovulation (21–42 day menstrual cycles with variation <8 days),
  - Male partner has had a normal semen analysis in the last five years according to WHO criteria [36] (volume ≥1.5 ml, progressive motility ≥32 %, concentration ≥15 million/ml) or total motile count >10 million
4. Have either:
  - Two ovaries and two probably patent fallopian tubes (normally confirmed by laparoscopy or hysterosalpingography (HSG))
  - A previous intrauterine pregnancy, and no subsequent surgery, infection or ectopic pregnancy that may reduce tubal patency
5. Body mass index (BMI) ≤ 35 kg/m^2^
6. Willing and able to have regular sexual intercourse in the cycle in which the procedure is performed, and for two cycles following the procedure.

##### PIP-PCOS

1. In a relationship where the couple is having regular, unprotected sexual intercourse and pregnancy is desired
2. 42 years of age or younger at the time of randomisation
3. Meet the Rotterdam criteria for PCOS, at least two of the following [37]:
  - Oligoovulation or anovulation (confirmed by luteal phase progesterone test or history of irregular cycles)
  - Excess androgen activity (elevated serum testosterone or clinical signs such as excess hair)
  - Polycystic ovaries (as evidenced on ultrasound)
4. Male partner has had a normal semen analysis in the last five years according to WHO criteria [36] (volume ≥1.5 ml, progressive motility ≥32 %, concentration ≥15 million/ml) or total motile count >10 million
5. Have either
  - Two ovaries and two probably patent fallopian tubes (normally confirmed by laparoscopy or HSG)
  - A previous intrauterine pregnancy, and no subsequent surgery, infection or ectopic pregnancy that may reduce tubal patency
  - Confirmed to have ovulated in a total of six or fewer ovulation induction cycles (as assessment of tubal patency may not be recommended until failure to achieve pregnancy following a number of cycles of successful ovulation)
6. Body mass index (BMI) ≤ 35 kg/m^2^
7. Willing and able to have regular sexual intercourse in the cycle in which the procedure is performed, and for two cycles following the procedure
8. Planning to undergo at least three consecutive cycles of ovulation induction (unless pregnancy occurs prior to completion of three study cycles).

#### Exclusion criteria

Women who meet the following exclusion criteria are not eligible to take part in the PIP studies:

##### PIP-IVF, PIP-PCOS and PIP-UE

1. Have had any disruptive instrumentation within the uterine cavity (for example, hysteroscopy, hysterosalpingography, laparoscopy, surgically managed miscarriage or endometrial biopsy) within three months prior to day one of the planned embryo transfer cycle/menstrual cycle/ovulation induction cycle, or planning to undergo a procedure involving disruptive instrumentation at any stage during the study. Embryo transfer and intrauterine insemination (IUI) procedures are not considered to cause disruptive instrumentation
2. Entered previously into this study or participated in another trial in the last 30 days
3. Any contraindication to endometrial biopsy and/or being pregnant and/or carrying a pregnancy to term.

##### Additional exclusion criteria for PIP-UE

4. On ovulation induction medication or any other fertility treatment or planning any fertility treatment during the study period
5. Have had a miscarriage in the last 12 months or have moderate or severe endometriosis (grade 3 or 4) and therefore do not meet the criteria for unexplained infertility [38]
6. Have recurrent miscarriage (spontaneous loss of three or more clinical pregnancies [39]).

##### Additional exclusion criteria for PIP-PCOS

1. The presence of any other cause of infertility, where spontaneous conception is unlikely (for example, large fibroids, moderate or severe endometriosis (grade 3 or 4) or severe male factor)
2. Have recurrent miscarriage (spontaneous loss of three or more clinical pregnancies [39])

### Standard care

Standard care will be identical in both arms of each PIP study. The PIP studies are pragmatic trials, and standard care may differ slightly between participating centres. For example, some centres may prefer an antagonist protocol and others may prefer a long course agonist protocol for IVF cycles.

Participants in PIP-IVF are either taking part in an IVF/ICSI cycle involving controlled ovarian stimulation, oocyte collection and fresh embryo transfer, or are planning a frozen-thaw embryo transfer. Any IVF protocol or use of additional treatments, such as pre-implantation genetic diagnoses or assisted hatching, is acceptable. Similarly, artificial, manufactured and natural cycles for frozen-thaw embryo transfers are acceptable.

PIP-PCOS participants are on ovulation induction medication: clomiphene, letrozole, follicle-stimulating hormone (FSH) or metformin. The choice of medications, dosages and combinations of medications may vary.

Participants in PIP-UE should not be on any fertility treatment. Participants in PIP-PCOS and PIP-UE are asked not to undergo any additional fertility treatment; for example, IUI and IVF are considered protocol violations. Vitamins, dietary supplements and other medications or procedures which are not normally considered fertility treatments, such as acupuncture, are permitted in all studies.

### Eligibility assessment

Potentially eligible women will be informed about the PIP study by invitation letter, advertisement (for example, poster or newsletter) or their treating clinician or nurse. All potential participants will have the opportunity to ask questions about the study and discuss any concerns or queries prior to making a decision about whether to take part. Informed consent will be obtained from all participants in writing prior to their recruitment. Informed consent will be undertaken by a PIP study researcher who is familiar with the study details and has adequate training and experience in trial recruitment (see Tables 1 and 2).

**Table 1:** Schedule of procedures and outcome measurements for PIP-IVF

	Pre-study	Day 1 of prior cycle	Day 3 of prior cycle-day 3 of IVF/embryo transfer cycle	Day after procedure	IVF cycle/embryo transfer	About 2 weeks after embryo transfer	About 6 weeks gestation	≥12 weeks gestation	Birth
Eligibility screen	x								
Informed consent	x								
Randomisation		x							
Endometrial scratching (intervention group only)			x						
Pain (VAS) (intervention group only)			x						
Adverse events (intervention group only)			x	x					
Bleeding (intervention group only)				x					
IVF cycle characteristics					x				
Biochemical pregnancy						x			
Clinical pregnancy							x		
Ongoing pregnancy								x	
Live birth									x

**Table 2:** Schedule of procedures and outcome measurements for PIP-UE and PIP-PCOS

	Pre-study	Day 1-12 of cycle 1	Day after procedure day	End of study/end of cycle 3	About 6 weeks gestation	≥12 weeks gestation	Birth
Eligibility screen	x						
Informed consent	x						
Randomisation		x					
Study intervention		x					
Pain (VAS)		x					
Adverse events		x	x				
Bleeding			x				
Cycle characteristics (e.g., intercourse frequency, ovulation medication dose (PIP-PCOS only), via study diary)				x			
Clinical pregnancy					x		
Ongoing pregnancy						x	
Live birth							x

The details of all women assessed for eligibility will be recorded to ensure reporting as per the CONSORT statement [40]. This record will include reasons for ineligibility or nonparticipation.

### Randomisation

A third-party, Internet-based, data collection and randomisation system will be used to confirm participant eligibility and ensure allocation concealment; that is, participant allocations are concealed within the system until the patient is randomised. Only participants who meet the eligibility criteria can be randomised. In instances where computer or Internet access is not reliable, participants will be randomised using sequentially numbered, opaque, sealed envelopes. Dedicated PIP study personnel will be responsible for randomising participants at each site. Participants will be randomised in a 1:1 ratio using block randomisation of two different sizes between 5–15 repeating in random order, stratified by recruiting clinic and by fresh or frozen-thaw embryo transfer (the latter in PIP-IVF only).

### Timing of randomisation and the study procedure

#### ᅟ

##### PIP-IVF

In PIP-IVF, eligible women will be randomised to the study on or after day one of the cycle that precedes the embryo transfer cycle (Table 1). For example, in participants scheduled for a long course buserelin cycle, randomisation will ideally take place on day one of the cycle, 21 days before down-regulation begins. Women will be informed of their allocation immediately after randomisation. Women randomised to the procedure group will be scheduled to undergo an endometrial biopsy procedure between day three of the cycle preceding embryo transfer and the third day of the embryo transfer cycle. Day one of the embryo transfer cycle is defined as either the day before the first day of controlled ovarian stimulation or day one of a menstrual period/withdrawal bleed for frozen-thaw embryo transfer cycles.

The large window of opportunity to perform the endometrial biopsy procedure is based on results from previous studies. Endometrial injury has been reported to have a beneficial effect as early as the menstrual period of the cycle preceding embryo transfer [20], and as late as the menstrual period following down-regulation [41]. This flexibility is also in keeping with the pragmatic nature of these studies.

##### PIP-PCOS and PIP-UE

Women who are eligible for PIP-UE or PIP-PCOS will be scheduled for the procedure between days one and 12 of a menstrual cycle (PIP-UE) or ovulation induction cycle (PIP-PCOS). The women will be randomised once they arrive at the clinic, allocating them to either undergo the endometrial biopsy or a sham procedure (Table 2).

The rationale behind timing the study procedure during the follicular phase of the cycle is threefold. Firstly, there is some concern that performing an intrauterine procedure in the luteal phase may disrupt a newly implanted embryo and therefore reduce the chance of pregnancy. Secondly, women would need to be asked to avoid conceiving during the pipelle biopsy cycle, and this would reduce the women’s overall opportunity to conceive. Lastly, it is thought that the endometrial biopsy procedure may be less uncomfortable and less likely to be abandoned due to inability to pass the pipelle if performed during the menstrual period, as the cervical canal is slightly more open. This is despite suggestion that endometrial injury induced decidulisation is more prominent in the luteal phase of the cycle [42].

The similarities and differences between the three PIP studies are outlined in Table 3.

**Table 3:** Comparison of the design of the three PIP studies

	PIP-IVF	PIP-UE	PIP-PCOS
Standard care	IVF/ICSI/embryo transfer	No treatment	Ovulation induction medication (metformin, clomiphene,
Method of attempted conception	IVF/ICSI/embryo transfer	Intercourse	Intercourse
Randomisation timing	Approx. day 1 of cycle prior to treatment cycle	Immediately prior to the intervention	Immediately prior to the intervention
Intervention	Endometrial scratching by pipelle	Endometrial scratching by pipelle	Endometrial scratching by pipelle
Control group	No procedure	Placebo procedure	Placebo procedure
Intervention timing	Between day of the cycle prior to IVF, until day 3 of the IVF cycle	Between days 1–12 of the first study cycle	Between days 1–12 of the first study cycle
Blinding	Open label	Single-blind (participants are blind)	Single-blind (participants are blind)
Number of study cycles/opportunities to conceive	One	Three	Three
Study diary	No	Yes	Yes

### Study procedures

A gynaecologist or trainee gynaecologist with experience in performing endometrial biopsies will undertake the endometrial biopsy and placebo procedures. Participants may be advised to attend with a full bladder and to take paracetamol prior to the procedure to minimise any discomfort.

#### Endometrial biopsy

Participants randomised to the intervention arm of the PIP studies will undergo an endometrial biopsy procedure, performed using a biopsy catheter (for example, Pipelle de Cornier, Laboratoire CCD, France).

**Fig. 1:**
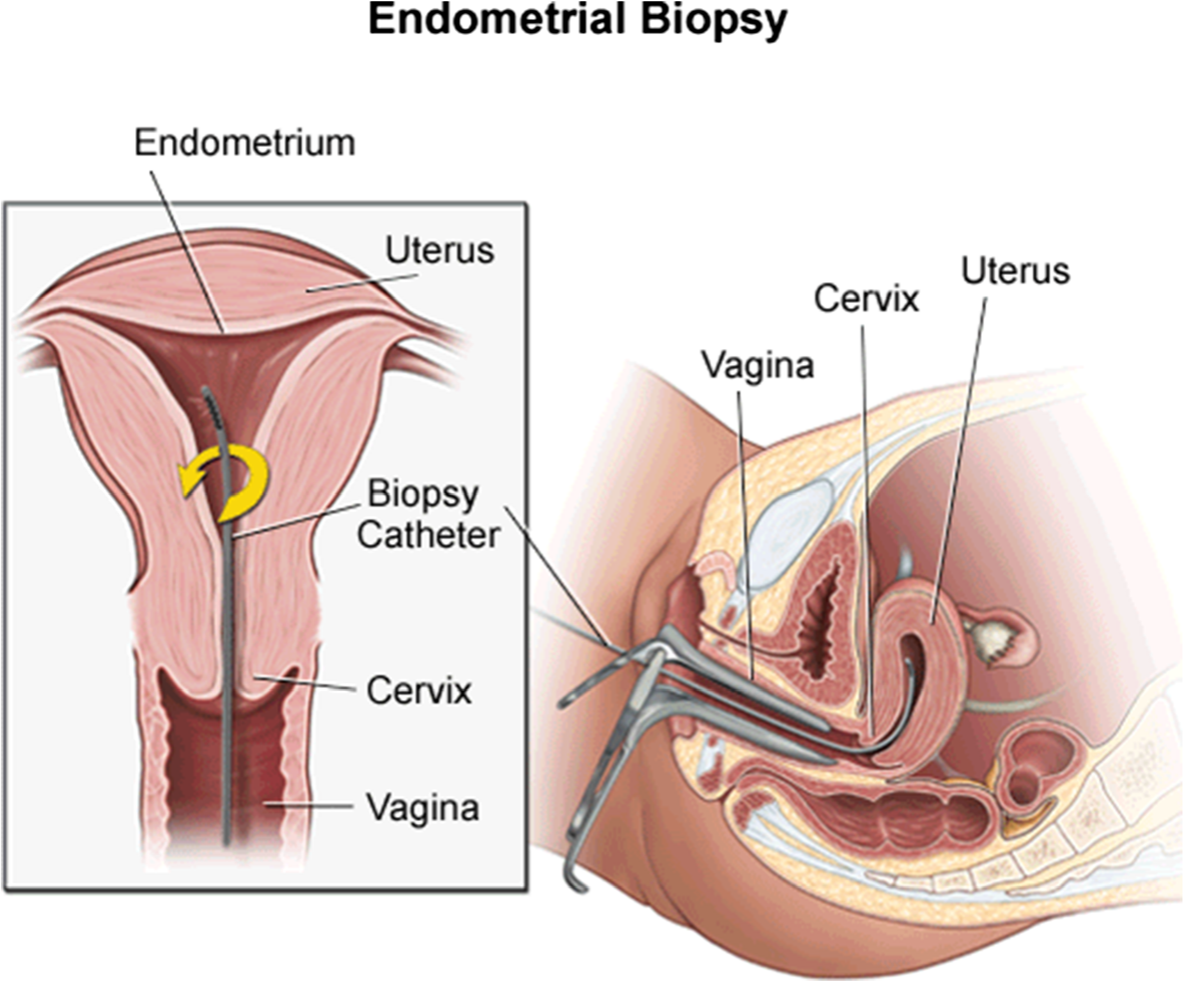
The endometrial biopsy procedure. Image provided by Krames Staywell, 780 Township Line Road, Yardley, PA 19067, 267-685-2500)

Unless otherwise requested, the woman will lie in the dorsal or lithotomy position and a speculum will be inserted into the vagina. The biopsy catheter will be inserted through the cervix up to the fundus. A tenaculum may be placed on the anterior lip of the cervix to help straighten the internal os to aid delivery. The internal piston is withdrawn to create negative pressure. The catheter is advanced to the fundus and then moved back and forth four times, rotating 90 degrees each time, to collect the biopsy (Fig. 1). The catheter is then removed followed by the speculum. The catheter containing the biopsy sample is then discarded unless the endometrial sample is required for another purpose.

If the pipelle is unable to be inserted into the uterus, local anaesthetic and cervical dilation are permitted, or a second attempt may be scheduled. The procedure is to be abandoned at the participants’ request — for example, due to pain or discomfort — or if the operator is unable to pass the pipelle. These participants will be retained in the group denominator as per intention-to-treat principles.

#### Placebo procedure

In PIP-PCOS and PIP-UE, women in the control arm of the trial will undergo a placebo procedure which is designed to blind participants to their allocation. If couples who are attempting to conceive with sexual intercourse are aware of their study allocation, they may purposely or inadvertently influence their pregnancy outcome through behavioural changes, such as a change in sexual frequency or timing.

For the placebo procedure the women will lie in the dorsal or lithotomy position and a speculum will be inserted into the vagina. The biopsy catheter will be placed at the posterior fornix but not inserted into the external os of the cervix. The internal piston is withdrawn and the catheter moved and rotated slightly to replicate the standard biopsy action. The catheter is then removed, followed by the speculum. The catheter is then discarded.

Participants randomised to the control group in PIP-IVF will not undergo any procedure that is additional to standard care.

Shortly following the procedure and before leaving the clinic, participants will be asked to record the pain they experienced during the procedure on a 10 cm VAS. Participants will also be handed an information leaflet detailing what to expect following an endometrial biopsy, and told to seek medical attention and contact the study coordinator if they experience any adverse events that are potentially associated with the procedure, for example, heavy bleeding or signs of infection such as foul smelling discharge.

In PIP-IVF, participants will be contacted within one week of the procedure and asked to report whether they had any vaginal bleeding the day following the procedure. Women in PIP-PCOS and PIP-UE will be asked to complete a study diary for the three study cycles. In this diary they will record the date of the procedure, whether they had any vaginal bleeding the day following the procedure, each day that they have their period and each time they have sexual intercourse during the on-study period. Women will continue to complete the study diary until their third period following the procedure or until confirmation of a clinical pregnancy (that is, all patients have three cycles or opportunities for conception within the on-study period). Participants should be contacted at least once a month to check in and ensure they are completing the study diary and to provide an opportunity to identify any protocol violations.

### Assessment of participant blinding

The sham procedure employed in PIP-UE and PIP-PCOS is designed to blind participants to their randomisation allocation. The sham procedure involves placing the pipelle in the posterior fornix and not inserting it through the cervix or into the womb, and hence it is expected to cause less pain on average than the interventional procedure. Although participants may not know how much pain to expect from either procedure, some participants may have prior expectations regarding the anticipated degree of pain from either procedure, and this may reduce the efficacy of the blinding. For example, women may have had previous intrauterine procedures or read information about endometrial biopsy on the Internet.

To assess whether participants in PIP-PCOS and PIP-UE are truly blinded to their study allocation, they will be asked to guess their study allocation (from the options of: pipelle procedure, placebo procedure or don’t know) and to supply a reason to support their guess. Participants will be randomised a second time following the procedure to either the ‘ask early’ group or the ‘ask late’ group. The ‘ask early’ group will be asked to guess their allocation within one week of the procedure, and the ‘ask late’ group will be asked to guess their allocation at the end of the study (that is, when the participant knows whether or not they have fallen pregnant in the study). Participants randomised to early will also be asked to guess again at the end of the study, to assess whether there are changes in response over time. Participants will be informed of their allocation after they guess their allocation at the end of the study.

### On-study period (study duration)

#### ᅟ

##### PIP-IVF

**Fig. 2:**
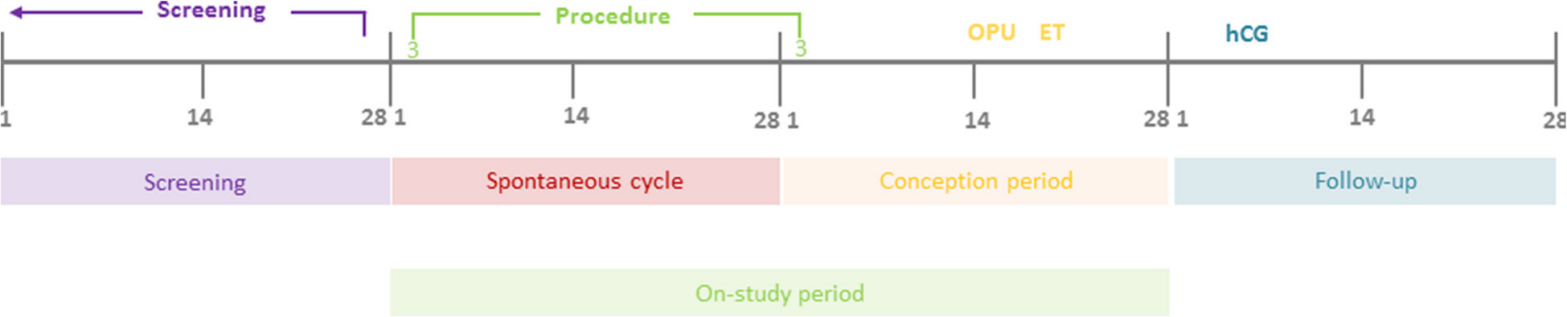
Timing of endometrial injury in PIP-IVF in relation to the IVF cycle and the on-study period. OPU = oocyte pick-up, ET = embryo transfer, hCG = human chorionic gonadotrophin (pregnancy) test

Women in PIP-IVF who undergo embryo transfer are usually scheduled for a serum human chorionic gonadotrophin test to confirm pregnancy approximately two weeks following embryo transfer (Table 1). Women with a positive pregnancy test will be followed up to determine clinical pregnancy by ultrasound at approximately six weeks gestation. The cycle prior to embryo transfer and the embryo transfer cycle are defined as the on-study period (Fig. 2). If the participants’ IVF cycle or embryo transfer is delayed, the recorded outcome will be the pregnancy result of the first embryo transfer which takes place within three calendar months of the expected day one of the embryo transfer cycle/day one of stimulation injections. The expected day one of the embryo transfer cycle is recorded at the time of randomisation.

##### PIP-PCOS and PIP-UE

**Fig. 3:**
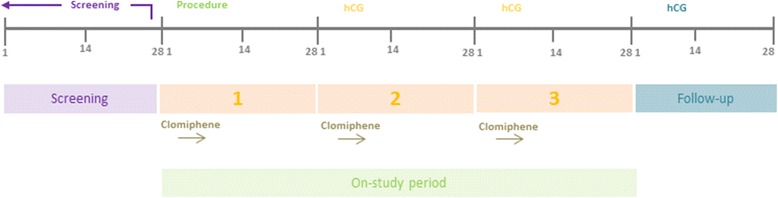
Timing of endometrial injury in PIP-PCOS in relation to the on-study period. hCG = human chorionic gonadotrophin (pregnancy) test

Participants in PIP-UE and PCOS are asked to try to conceive for three cycles: the cycle in which they undergo the study procedure and the two following cycles. Participants remain in the study until they either achieve a clinical pregnancy or get their third menstrual period following the procedure. This means that each participant has three cycles whilst on the study where they have the opportunity to conceive. For PIP-PCOS women this time frame is constant regardless of whether the cycles are ovulatory or not. The three study cycles are defined as the on-study period (Fig. 3).

### Follow-up

The first clinical outcome assessment (clinical pregnancy) will be done via ultrasound at approximately six weeks gestation at the respective trial centres. Ongoing pregnancy will then be assessed via ultrasound on or after 12 weeks gestation. Live birth is the primary study outcome and will be determined via correspondence with participants, from hospital records or via outcome data collected retrospectively at the trial centres.

### Statistics

#### Sample size

##### PIP-IVF

Based on the results of a Cochrane review on endometrial injury for IVF, a difference between arms of 16 % may be expected (pooled live birth rates of 33 % and 17 % [43]). At 90 % power and a significance level of 5 % in a two-sided test, at least 188 women are required in each arm (376 in total). In order to power for detection of any subgroup differences (for example, whether endometrial injury increases live birth rate in women undergoing frozen-thaw transfers) and assuming an equal recruitment of fresh and frozen transfer, the required sample was doubled, raising the total number required to 752.

##### PIP-UE

Current rates of pregnancy in women with unexplained infertility undergoing expectant management are approximately 3 % per cycle [44]. An expected increase in pregnancy rates to 9 % in the biopsy arm might be expected based on previous studies. With 3 months of expectant management, this equates to an expected cumulative difference of 25 % vs 9 % in the biopsy and sham arms respectively. With an adjustment for live birth rates, which are generally 19 % lower than pregnancy rates [45], the expected difference becomes 20 % vs. 7 %. At 90 % power and a significance level of 5 % in a two-sided test, 158 women are required in each arm (316 in total).

##### PIP-PCOS

PIP-PCOS sample size is based on the PCOS I trial, where a live birth rate per visit in the clomiphene arm was 5 % for the first 3 months [46]. An increase to 12 % per cycle as per previous studies might be expected. At 90 % power and a significance level of 5 % in a two-sided test, 126 women are required in each arm (252 in total).

Applying a 10 % contingency in each study to account for 1) women allocated to the biopsy group who fail to have a biopsy due to difficulty passing the pipelle, and 2) for participant attrition, the following numbers of participants are required in each study: PIP-IVF = 840, PIP-UE = 350, PIP-PCOS = 280. In the context of growing uncertainty regarding the beneficial effects of endometrial scratching, a power of 90 % was selected to reduce the probability of a type 2 error.

### Statistical analysis

Demographic and baseline data will be compared between the two groups using the chi-squared test or *t*-test. Outcome data for the primary analyses will be treated in an intention-to-treat manner, such that all participants remain in the groups to which they were randomised. If no outcome data is available for the primary outcome of live birth, this will be imputed to assume that women with no outcome data did not have a live birth. Sensitivity analyses based on different assumptions may be used for secondary analyses. Per protocol analyses will be conducted as a secondary analysis.

For binary outcomes (for example, live birth), risk ratios will be calculated with associated 95 % confidence intervals and *p* values. ANOVA or logistic regression analysis will be used for the analysis of pre-specified subgroup effects. For continuous outcomes (such as pain), the mean difference will be calculated and the student’s *t*-test will be used.

Assessment of participant blinding will be performed using both James’ and Bang’s blinding indices [47, 48]. All statistical analysis will be performed using STATA or a similar statistical programme.

### Subgroup analyses

The following subgroup analyses are planned for the primary outcome of live birth:

#### ᅟ

##### PIP-UE

1. Type of embryo transfer (fresh or frozen)
2. Number of embryos transferred (single or double)
3. Number of previous embryo transfer procedures not resulting in a clinical pregnancy (for example, 0–1, 2–4, ≥5)
4. Number of embryos transferred previously not resulting in a clinical pregnancy (for example, 0–1, 2–4, ≥5)
5. Timing of endometrial biopsy (for example, follicular phase: cycle prior to embryo transfer cycle, luteal phase: cycle prior to ET cycle, follicular phase: embryo transfer cycle)
6. Major cause of infertility (for example, male factor, endometriosis, ovulatory factor, unexplained, tubal factor, other)
7. History of endometriosis (endometriosis or no endometriosis)
8. Type of IVF (IVF or ICSI)
9. Type of stimulation protocol (for example, long agonist, ultralong agonist, short agonist, antagonist)
10. Duration of infertility (for example, < 2 years, 2–5 years, >5 years)
11. Stage of embryos at transfer (for example, day 5/6 or day 2/3)

##### PIP-PCOS

1. The type of ovulation induction medication the participant was on when they conceived (for example, clomiphene, letrozole, metformin, FSH and each combination used)
2. Number of previous ovulatory ovulation induction cycles (for example, 0–2, 3–6, >6)
3. Presence of endometriosis (endometriosis or no endometriosis)
4. Timing of endometrial injury (for example, during menstruation or not)

##### PIP-UE

1. Presence of endometriosis (endometriosis or no endometriosis)
2. Timing of endometrial injury (for example, during menstruation or not)
3. Duration of infertility (for example, < 2 years, 2–5 years, >5 years)

For the outcome of pain during the procedure the following subgroup analysis will be performed:

1. Previous cervical surgery (yes or no)
2. Whether a tenaculum was used during the procedure (yes or no)
3. Whether the procedure was performed during menstruation (yes or no).

Subgroup analyses will also be conducted to assess whether the probability of completing the endometrial biopsy procedure (that is, not abandoning the procedure) or needing a tenaculum to complete the procedure, is associated with the age of the woman or whether she had previous cervical surgery.

Any subgroup analyses conducted which are not pre-specified will be clearly stated as ad hoc and will be used for hypothesis generating only.

### Study sites and recruitment

The PIP trials are international, multi-centre trials with recruitment currently underway in a total of ten sites across six countries, as of January 2016 (Table 4). Recruitment at additional sites may commence in the future. It is anticipated that recruitment will be completed by mid-2017.

**Table 4:** Recruiting centres

Recruiting centre	Country
Fertility Plus, Auckland	New Zealand
Repromed, Auckland	New Zealand
Fertility Associates, Wellington	New Zealand
Fertility Associates, Christchurch	New Zealand
Fertility Associates, Auckland	New Zealand
Sahlgrenska University Hospital, Gothenburg	Sweden
Leuven Fertility Centre, Leuven	Belgium
Royal Women’s Hospital, Melbourne	Australia
Medical School of Ribeirao Preto, Sao Paulo	Brazil
El-Khayat Clinic, Cairo	Egypt

### Ethics and confidentiality

At the time of submitting this Protocol the PIP studies had received ethical approval from: 1) Northern A Health and Disability Ethics Committees, Ministry of Health, New Zealand, 2) Research and Human Research Ethics Committee, The Royal Women’s Hospital, Melbourne, Australia, 3) Research Ethics Committee, Faculty of Medicine, Cairo University, Egypt, 4) Ethics Committee of the Hospital of the Clinical Medical College, Riberio Preto, Brazil, 5) Institutional Ethics Committee, Queensland Fertility Group, Australia, 6) Regional ethical review board in Gothenburg, 7) Committee Medical Ethics, UZ KU Leuven/Research. All relevant ethics and locality approvals have been obtained prior to recruitment commencing at each participating site, and will be obtained prior to recruitment at any site that starts recruiting in the future.

All patient health information will be treated confidentially. Investigators and study staff will only have access to health information about participants recruited at their clinic; for example, site A will not have access to patient data from site B.

The PIP studies will be conducted according to the Principles of Good Clinical Practice as defined in the Medicines for Human Use (Clinical Trials) Amended Regulations 2006.

### Roles and responsibilities

The Chief Principal Investigator is Professor Cynthia Farquhar, based at Fertility Plus, Auckland District Health Board, which is the coordinating site for the PIP studies. The coordinating centre is responsible for monitoring recruitment rates, communicating protocol changes to participants, investigators and clinical trial registries, and conducting data quality and completeness auditing. Together the Chief Investigator and at least one investigator from each recruiting site form the Steering Committee for the PIP studies. The role of this committee is to consider matters relating to protocol amendments and recruitment strategies. The results of the PIP studies will be published in peer-reviewed journals within 12 months of trial closure, and be uploaded to the trial registration website, as per WHO recommendations [49]. Authorship of any resulting publications will be governed by the Steering Committee. The results will also be made available to trial participants who indicated they wished to receive a trial report on their consent form.

The PIP trials are investigator initiated studies, and no study sponsor is applicable. Provisions relating to compensation for any adverse outcome associated with trial participation are organised on a case-by-case basis at the recruiting site.

### Participant withdrawal

Participants are free to withdraw from the study at any time. Prior to randomisation, participants will give their consent that any information collected about them, up to the point that they withdraw, can still be processed and analysed in the study. If a participant chooses to withdraw from the study, from this point onwards no further study procedures may occur and no further data may be collected. There is no replacement of participants who withdraw, as a contingency for this has been built into the sample size calculations.

### Protocol violations

Participants who do not follow the planned protocol are considered to have a protocol violation. This includes, for example, those for whom an endometrial biopsy is not successful or those who undergo an additional fertility treatment. The nature of the protocol violation will be documented for each participant. Participants who withdraw or who have a protocol violation will remain in the study denominator as per intention-to-treat principles.

### Interim analyses and data and safety monitoring

No interim analyses will be conducted. No data safety monitoring committee will be established.

Adverse events are defined as any untoward medical occurrence in a randomised patient regardless of its causal relationship to study treatment. Provision has been made for actively recording adverse events which are expected, for example, miscarriage, multiple gestations and pain during the procedure. Additionally, due to concern regarding the possible influence of endometrial disturbance on the implantation site of an embryo, the location of the placenta will be recorded following live birth.

Participants will be asked to report any other adverse events to the study coordinator when they occur; these will not be actively recorded. All adverse events that occur during the study period will be documented on a designated data form. It should be noted that endometrial biopsy is a safe and well-tolerated procedure which is routinely performed in women in whom uterine pathology is suspected, for example, women with menorrhagia. Adverse events and complications related to this procedure include infection, haemorrhage and perforation of the uterus, but these are rare [6, 7].

### Data collection

Data will be entered and stored electronically on the secure online PIP studies data collection and randomisation system. This database has inbuilt logical and validation, such that only impossible values are not allowed and to avoid cases of missing data where it is not applicable. Some information will be retrieved from source documents such as patient notes, and other clinical information may be entered directly into the PIP database. Individual logins will be issued to each person, and individuals will only have access to data regarding participants who are recruited at their clinic.

Researcher assistants, doctors and nurses involved in the PIP study will be responsible for the data collection at each site. Study data will be collected at numerous time points during the study journey (Table 1, Table 2). Data may be collected directly for the purposes of the study, for example, the pain score as measured following the procedure or the intercourse frequency as recorded in the diary, or indirectly via the patient records which are routinely recorded for their treatment purposes (for example, date of embryo transfer).

### Data storage

The PIP database is hosted by the University of Auckland, and will run on a dedicated server at the University of Auckland server farm, behind firewalls with daily backup and 128-bit SSL encryption. The University of Auckland will retain ownership of the PIP studies trial data. Data will be stored securely on the PIP database for 10 years following the completion of the study, after which time it will be disposed.

## Discussion

IVF is the leading treatment for subfertility; however, the success rate remains modest at approximately 30 % per cycle [2, 3]. Endometrial scratching is currently being suggested as a technique to improve the probability of embryo implantation and therefore pregnancy in women undergoing IVF. However, due to the potential for bias associated with many of the available studies, the presence of a therapeutic effect remains uncertain.

The PIP trials are designed to address the gaps in the utility of endometrial scratching as a treatment for subfertility in three different populations. If the beneficial effect of this intervention can be confirmed in these settings, endometrial scratching will provide a cost-effective method for helping women and couples to conceive. On the other hand, if endometrial scratching is found to have no effect, or to be detrimental to the probability of conception, the use of this procedure can be abandoned amongst the clinics and doctors who are recommending this procedure to their patients.

### Trial status

Ongoing (recruitment commenced June 2014).

## Abbreviations

CI: confidence interval
FSH: follicle-stimulating hormone
ICSI: intracytoplasmic sperm injection
IUI: intrauterine insemination
IVF: in vitro fertilisation
PCOS: polycystic ovarian syndrome
PIP: Pipelle for Pregnancy
RIF: recurrent implantation failure
UE: unexplained infertility
VAS: visual analogue scale
